# Osteoporosis guidelines on TCM drug therapies: a systematic quality evaluation and content analysis

**DOI:** 10.3389/fendo.2023.1276631

**Published:** 2024-01-22

**Authors:** Luan Zhang, Jiahui Li, Runsheng Xie, Lingfeng Zeng, Wenjia Chen, Hui Li

**Affiliations:** ^1^ The Second Clinical College of Guangzhou University of Chinese Medicine, Guangzhou, China; ^2^ The Second Affiliated Hospital of Guangzhou University of Chinese Medicine, Guangzhou, China; ^3^ Research Team of Chinese Medicine Standardization, The Second Affiliated Hospital of Guangzhou University of Chinese Medicine, Guangzhou, China; ^4^ Research Team of Chinese Medicine Standardization, Guangdong Provincial Hospital of Chinese Medicine, Guangzhou, China; ^5^ State Key Laboratory of Traditional Chinese Medicine Syndrome, The Second Affiliated Hospital of Guangzhou University of Chinese Medicine, Guangzhou, China

**Keywords:** guidelines, osteoporosis, TCM drug therapies, quality evaluation, stratified analysis, recommendations

## Abstract

**Objective:**

The aims of this study were to evaluate the quality of osteoporosis guidelines on traditional Chinese medicine (TCM) drug therapies and to analyze the specific recommendations of these guidelines.

**Methods:**

We systematically collected guidelines, evaluated the quality of the guidelines using the *Appraisal of Guidelines Research and Evaluation* (AGREE) II tool, and summarized the recommendations of TCM drug therapies using the Patient-Intervention-Comparator-Outcome (PICO) model as the analysis framework.

**Results and conclusions:**

A total of 20 guidelines were included. Overall quality evaluation results revealed that four guidelines were at level A, four at level B, and 12 at level C, whose quality needed to be improved in the domains of “stakeholder involvement”, “rigor of development”, “applicability” and “editorial independence”. Stratified analysis suggested that the post-2020 guidelines were significantly better than those published before 2020 in the domains of “scope and purpose”, “stakeholder involvement” and “editorial independence”. Guidelines with evidence systems were significantly better than those without evidence systems in terms of “stakeholder involvement”, “rigor of development”, “clarity of presentation” and “applicability”. The guidelines recommended TCM drug therapies for patients with osteopenia, osteoporosis and osteoporotic fracture. Recommended TCM drugs were mainly Chinese patent medicine alone or combined with Western medicine, with the outcome mainly focused on improving bone mineral density (BMD).

## 1 Introduction

Osteoporosis is a systemic skeletal disease characterized by decreased bone mass and microarchitectural deterioration of bone tissue, leading to increased bone fragility and fracture risk (1). An estimated 200 million people worldwide suffer from osteoporosis (2), and osteoporotic fractures account for 34.8% of the global noncommunicable disease burden worldwide (3). In Europe, over 230,000 men and women are at high risk of osteoporotic fractures (4), which account for more Disability Adjusted Life Years (DALYs) lost than common cancers (excluding lung cancer) (3). Additionally, osteoporosis and osteoporotic fractures cost European healthcare systems more than EUR 5.6 billion per year (4). It is estimated that the annual direct medical costs of osteoporosis-related fractures in the United States will reach $2.5 billion by 2025 (5).

As a treatment for osteoporosis, TCM drug therapies have garnered increasing attention (6). Studies have shown that TCM has a better pain-relieving effect, a higher total effective rate, and a lower risk of adverse events compared with regular Western medicine treatment (7, 8). With the widespread use of TCM drug therapies for osteoporosis, there is an urgent need for guidance on the best available methods. Thus, osteoporosis guidelines on TCM drug therapies were published, but their quality varied. Different guidelines even gave inconsistent recommendations (9–11). Additionally, the main treatment plan, indications, and efficacies of TCM still remain unknown.

Therefore, the study aimed to systematically collect relevant osteoporosis guidelines on TCM drug therapies, evaluate them using the *Appraisal of Guidelines Research and Evaluation* (AGREE) II tool, and analyze the specific factors that affect the quality of the guidelines. The ultimate goal was to provide a reference for future osteoporosis guideline development. Meanwhile, this study summarized the high-quality recommendations of TCM drug therapies in the guidelines, providing up-to-date and essential information for clinical decision-making.

## 2 Materials and methods

### 2.1 Literature acquisition

#### 2.1.1 Search strategy

A comprehensive search was conducted using PubMed, EMBASE, Web of Science, CBM, CNKI, VIP, and Wanfang Database to search for osteoporosis guidelines that included TCM drug therapies. To search guidelines repositories and grey literature, we used the National Institute for Health and Care Excellence (NICE), Guidelines International Network (G-I-N), World Health Organization (WHO), Scottish Intercollegiate Guidelines Network (SIGN), Medlive and Google Scholar. All relevant articles from inception to June 2023 were retrieved.

Search terms included: Osteoporosis, Bone Loss, Guideline, Guidance, Recommendation, etc. The full search strategy is presented in Appendix 1.

#### 2.1.2 Inclusion criteria and exclusion criteria

The inclusion criteria included: (1) published guidelines related to osteoporosis; (2) written in Chinese and English; (3) guidelines on TCM drug therapies, such as herbs, herbal extracts, TCM prescriptions, Chinese patent medicines, etc.

The exclusion criteria included: (1) guidelines for non-pharmaceutical TCM therapies, such as acupuncture, moxibustion, etc.; (2) outdated guidelines that have been replaced; (3) Chinese translations, adaptations, abstracts or overviews, and other interpretations of international guidelines, evaluation reports, etc.; (4) duplicate literature.

#### 2.1.3 Literature screening and data extraction

Two researchers (LZ, JL) independently screened the literature and extracted the basic information of the included guidelines (e.g., title, publication year, main developing organization, fund, etc.). In case of disagreement, the decision was taken by a third researcher.

### 2.2 Quality evaluation

#### 2.2.1 Overall evaluation of AGREE II

Two reviewers independently evaluated the 20 included guidelines using the guideline quality assessment tool AGREE II, which comprises six domains (scope and purpose, stakeholder involvement, rigor of development, clarity of presentation, applicability and editorial independence), including 23 items. The minimum score for each item is one point and the maximum score is seven points. The higher the score, the more consistent the content of the guideline with the requirements of the item. Score of each domain of a guideline is the percentage of the sum of the points of all items in that domain to the highest possible score in the domain (12).

According to the scores of the six domains, a guideline was classified into three levels: if six domains scored ≥60%, the guideline would be classified as A (recommended); if a guideline scored between 30% and 60% in three or more domains, it would be classified as B (recommended after modification and improvement); if a guideline had scores of ≤30% in three or more domains, it would be classified as C (not recommended).

Descriptive statistical analysis was then performed by calculating the total score of each domain, presented as mean ± standard deviation (SD).

The two independent samples T-test (using SPSS 26.0 software) was used to test consistency, which was calculated using the intraclass correlation coefficient (ICC). The degree of consistency (ICC) is classified according to the following criteria (13): poor (<0.40), fair (0.40–0.59), good (0.60–0.74) or excellent (0.75–1.00).

#### 2.2.2 Stratified analysis

Guidelines were stratified according to the following stratification factors: (1) publication year, either before or after 2020; (2) availability of funding; (3) whether an evidence system was used or not, and (4) whether they were developed by an organization (a society or an association) or by an individual or expert group. After stratification, a category wise statistical comparison of the scores of all domains were calculated (*P*<0.05).

### 2.3 Content analysis

Content analysis on the recommendations of TCM drug therapies in A- and B-level guidelines followed these two steps: (1) remove recommendations with incomplete information, low- and very low-quality evidence, before extracting the remaining medium- and high-quality recommendations; (2) summarize and analyze the extracted recommendations using the PICO model as an analysis framework.

## 3 Result

### 3.1 Literature screening and data extraction

**Figure 1:**
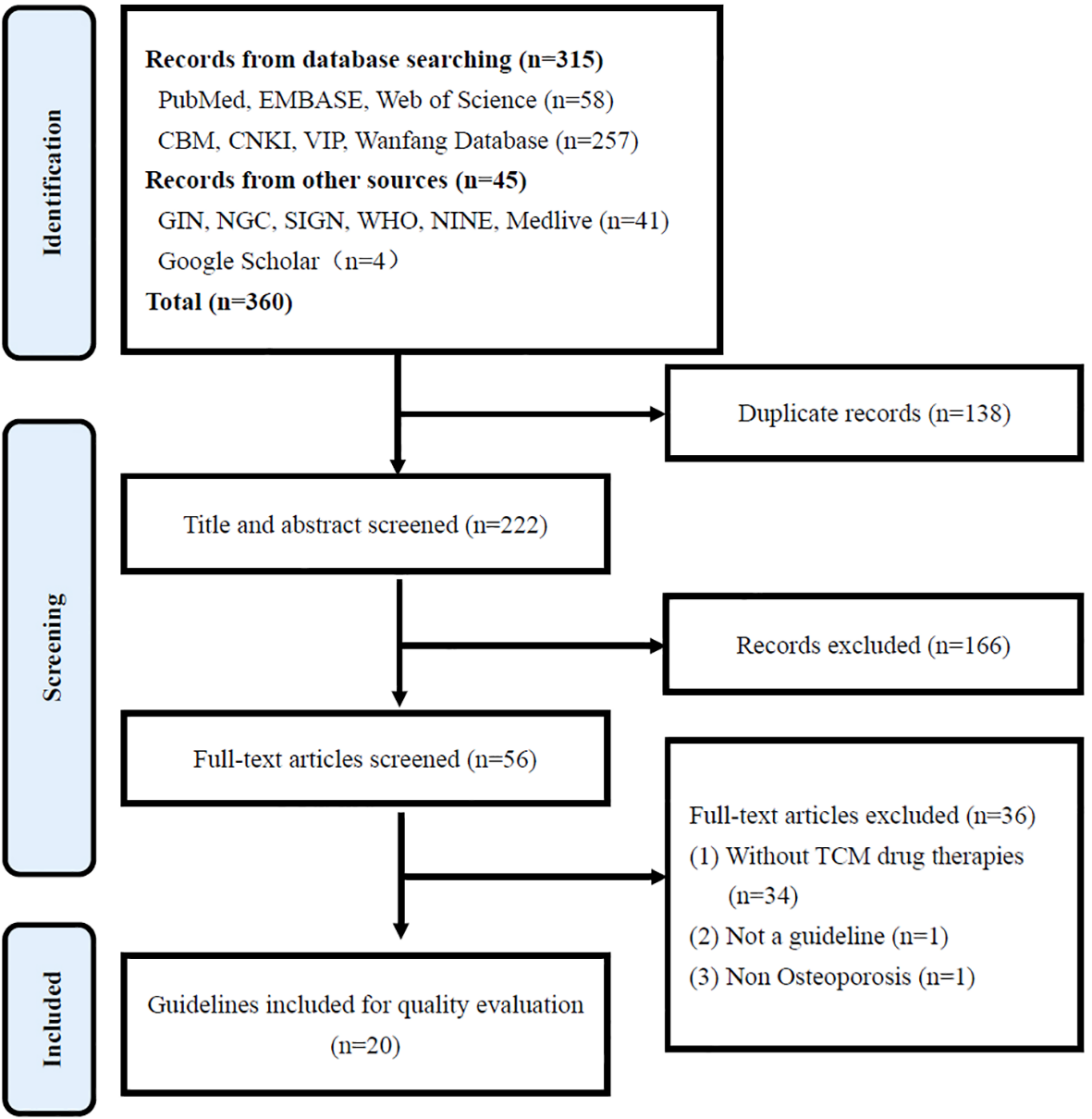
PRISMA flowchart for the selection of guidelines.

The search obtained 360 records, of which 138 were duplicates. According to the inclusion and exclusion criteria, 166 articles were excluded during the title abstract screening, 36 articles were removed during full-text screening, and 20 guidelines were finally included. The *Preferred Reporting Items for Systematic Reviews and Meta Analyses* (PRISMA) flowchart for the selection of guidelines is shown in **Figure 1**, and the basic characteristics of the included guidelines are shown in **Table 1**.

**Table 1:** The basic characteristics of the guidelines.

GN	Title	ES	Year	Fund	Organization /Group
1	Expert Consensus on Clinical Application of Gukang Capsule in Treatment of Osteoporosis (14)	GRADE	2022	R	CACM
2	Clinical practice guideline for postmenopausal osteoporosis with traditional Chinese medicine (9)	GRADE	2021	R	CACMS
3	Clinical Application Guidance for Treating Osteoporosis by Chinese Patent Medicine (2021) (10)	GRADE	2021	R	SP
4	Guidelines for TCM Diagnosis and Treatment of Postmenopausal Osteoporosis(2019 edition) (11)	GRADE	2020	NR	CACM
5	2018 China guideline for diagnosis and treatment of senile osteoporosis (15)	GRADE	2018	NR	CAGG
6	Clinical Practice Guideline of Traditional Medicine for Primary Osteoporosis (16)	TMES	2011	R	WHO
7	Expert Consensus on the Diagnosis and Treatment of Osteoporosis with Integrated Traditional Chinese and Western Medicine (17)	NR	2023	NR	WFCMS et al.
8	Expert Consensus on Community Management of Diagnosis and Treatment of Osteoarthritis and Osteoporosis in the Elderly (Version 2023) (18)	NR	2023	R	BMA
9	Sarcopenia osteoporosis expert consensus (19)	NR	2022	NR	CHPF
10	Guidelines for the Diagnosis and Treatment of Primary Osteoporosis (2022) (20)	NR	2022	NR	CSOBMR
11	Expert consensus on the diagnosis and treatment of osteoporosis in primary medical institutions (2021) (21)	NR	2021	NR	CHPF
12	Expert consensus on diagnosis and management of osteoporosis in geriatric hip fractures (22)	NR	2021	R	BPWF et al.
13	Expert consensus on prevention and treatment of osteoporosis in perimenopausal and postmenopausal women (23)	NR	2020	R	CAGG
14	Guideline for diagnosis and treatment of osteoporosis in men (24)	NR	2020	NR	CSOBMR
15	Traditional Chinese Medicine Expert Consensus on the prevention and treatment of primary osteoporosis (2020) (25)	NR	2020	NR	CAGG
16	Guidelines for diagnosis and treatment of osteoporosis with integrated traditional Chinese and Western medicine (26)	NR	2019	NR	CAITWM
17	Expert consensus of TCM hierarchical diagnosis and treatment of primary osteoporosis in Zhejiang Province (2017) (27)	NR	2018	R	CAITWMZP
18	Expert consensus on muscle, bone and osteoporosis (28)	NR	2016	NR	CAGG
19	Guidelines for the Prevention and Treatment of Diabetic Metabolic Bone Disease (29)	NR	2011	R	CACM
20	Alternative therapies for osteoporosis (30)	NR	2006	R	IARASM

GN, Guideline Number; ES, Evidence System; GRAGE, Grading of Recommendations, Assessment, Development, and Evaluations; TMES, Traditional Medicine Evidence System suggested by Jianping Liu; R, Repot; NR, Not Report; CACM, China Association of Chinese Medicine; CACMS, China Academy of Chinese Medical Sciences; SP, Standardization Project of Clinical Application Guidelines for the Treatment of Superior Diseases with Chinese Patent Medicines; CAGG, Chinese Association of Gerontology and Geriatrics; WHO, World Health Organization; WFCMS, World Federation of Chinese Medicine Societies; BMA, Beijing Medical Association; CHPF, China Health Promotion Foundation; CSOBMR, Chinese Society of Osteoporosis and Bone Mineral Research; BPWF, Bethune Public Welfare Foundation; CAITWM, Chinese Association of Integrated Traditional and Western Medicine; CAITWMZP, Chinese Association of Integrated Traditional and Western Medicine in Zhejiang Province; IARASM, Institute for Advanced Research in Asian Science and Medicine.

### 3.2 Quality evaluation

#### 3.2.1 Overall evaluation of AGREE II

The two reviewers independently evaluated the included guidelines with AGREE II. The ICC were between 0.878~0.982, indicating a high consistency. Among the 20 guidelines, four were recommended at level A (recommended), four were recommended at level B (recommended after modification), and 12 were recommended at level C (not recommended). Consistency and quality evaluation results are shown in **Table 2**.

**Table 2:** AGREE II domain scores and consistency tests for guidelines.

GN	AGREE II domain (%)						DN of Score ≥60% (n)	DN of Score ≤30% (n)	ICC (95%CI)	Level
	Scope and purpose	Stakeholder involvement	Rigor of development	Clarity of presentation	Applicability	Editorial independence				
1	91.67	75.00	60.41	100.00	62.50	95.83	6	0	0.947(0.880,0.977)	A
2	73.53	50.00	47.92	72.22	16.67	41.67	2	1	0.974(0.940,0.989)	B
3	88.89	77.78	89.58	100.00	60.42	100.00	6	0	0.931(0.845,0.970)	A
4	58.33	38.89	50.00	94.44	50.00	8.33	1	2	0.944(0.869,0.976)	B
5	83.33	77.78	79.17	100.00	77.08	62.50	6	0	0.950(0.887,0.978)	A
6	41.67	33.33	76.04	77.78	58.33	50.00	2	0	0.936(0.846,0.973)	B
7	80.55	58.33	31.25	69.44	35.42	79.17	4	1	0.952(0.851,0.982)	B
8	94.44	75.00	61.46	97.22	64.58	91.66	6	0	0.953(0.890,0.980)	A
9	58.33	47.22	7.29	55.56	12.50	29.17	0	3	0.960(0.908,0.983)	C
10	41.67	11.11	4.17	88.89	16.67	29.17	1	4	0.878(0.734,0.947)	C
11	58.33	63.89	8.33	58.33	25.00	25.00	1	3	0.953(0.894,0.980)	C
12	69.44	36.11	21.88	91.67	8.33	29.17	2	3	0.974(0.939,0.989)	C
13	58.33	33.33	21.88	72.22	8.33	29.17	2	3	0.973(0.938,0.988)	C
14	63.89	19.44	11.46	91.67	33.33	0.00	2	3	0.925(0.834,0.967)	C
15	66.67	36.11	6.25	61.11	22.92	8.33	2	4	0.939(0.857,0.974)	C
16	58.33	25.00	11.46	83.33	29.17	29.17	1	4	0.965(0.921,0.987)	C
17	58.33	47.22	8.33	66.67	25.00	25.00	1	3	0.982(0.953,0.993)	C
18	63.89	41.67	7.29	58.33	14.58	29.17	1	3	0.971(0.933,0.987)	C
19	27.78	13.89	0.00	50.00	0.00	37.50	0	4	0.967(0.925,0.986)	C
20	55.56	13.89	22.92	91.67	37.50	0.00	1	3	0.976(0.944,0.990)	C
Mean	64.65	43.75	31.35	79.03	32.92	40.00				
SD	17.25	21.89	28.71	16.85	22.20	30.69				

GN, Guideline number; DN, domain number.

##### 3.2.1.1 Scope and purpose

Mean score for the “scope and purpose” domain was 64.65% (SD 17.25%). While the guidelines clearly described their overall purpose, covered health issues and the target population, they rarely expressed health issues in the form of the PICO.

##### 3.2.1.2 Stakeholder involvement

Mean score for “stakeholder involvement” was 43.75% (SD 21.89%). Most of the guidelines clearly indicated the users of the guidelines, providing names, institutions, and regions of the members of the expert group, but did not collect the preferences and views of the target population. Descriptions of staffs responsibilities and research fields were incomplete.

##### 3.2.1.3 Rigor of development

The “rigor of development” domain, which is the most important area for guideline development, had the lowest average score of 31.35% (SD 28.71%). Five guidelines (10, 14–16, 18) scored over 60% in this domain, indicating a rigorous development process. However, most guidelines failed to use systematic search methods to retrieve evidence, establish clear evidence selection criteria, undergo external expert review, and provide specific updates.

##### 3.2.1.4 Clarity of presentation

The “clarity of presentation” domain had the highest average score of 79.03%, and the lowest SD of 16.85%. The recommendations given by the guidelines were clear and unambiguous, and different options were given for different clinical scenarios. However, some important recommendations were not presented in forms such as tables, special fonts, or flowcharts to enhance readability.

##### 3.2.1.5 Applicability

Mean score for “applicability” was 32.92% (SD 22.20%). The scores in this area were low. Most guidelines failed to address barriers and facilitators, or potentially related resources and other issues for guideline application, or to provide supporting tools for implementation.

##### 3.2.1.6 Editorial independence

Mean score for “editorial independence” was 40.00% (SD 30.69%). This domain requires that conflicts of interest among members of the guideline development team are documented and disclosed so that the content of the guidelines is not influenced by the sponsorship. Half of the guidelines reported sponsors and conflicts of interest, but did not adequately explain their role on guideline development.

#### 3.2.2 Stratified analysis

There were statistically significant differences in the “scope and purpose”, “stakeholder involvement” and “editorial independence” domains when comparing guidelines published before and after 2020 (*P*<0.05). Guidelines published after 2020 were found to be better than those before 2020. Additionally, guidelines with evidence systems significantly outscored those without in terms of “stakeholder involvement”, “rigor of development”, “clarity of presentation”, and “applicability” (*P*<0.05). Besides, there was no statistically significant difference in all domains of AGREE II in terms of whether the guideline was funded or not, and whether the development was by an association or an individual or expert group. Stratified analysis results are shown in **Table 3**.

**Table 3:** Stratified analysis results of the AGREE II domain scores of guidelines.

Stratification factors	Scope and purpose	Stakeholder involvement	Rigor of development	Clarity of presentation	Applicability	Editorial independence
publication year						
After 2020(n=9)	72.98	54.94	36.92	81.48	33.57	57.87
2020 and before(n=11)	57.83	34.60	26.80	77.02	32.38	25.38
P values	0.047 *	0.035 *	0.448	0.570	0.910	0.014 *
Fund						
NR(n=10)	63.33	41.94	21.67	76.11	31.67	30.00
R(n=10)	65.96	45.56	41.04	81.95	34.17	50.00
P values	0.743	0.723	0.135	0.454	0.809	0.150
Evidence system						
NR(n=14)	61.11	37.30	16.00	74.01	23.81	31.55
R(n=6)	72.90	58.80	67.19	90.74	54.17	59.72
P values	0.167	0.040 *	<0.001 *	0.038 *	0.002 *	0.057
Development institution						
Association(n=17)	63.23	43.14	27.45	77.45	31.98	38.73
Individual and expert group(n=3)	72.66	47.22	53.47	87.96	38.20	47.22
P values	0.397	0.774	0.153	0.332	0.667	0.670

NR, Not report; R, Report; *The difference was statistically significant.

### 3.3 Drug recommendations

After removing recommendations for TCM drug therapies with incomplete information, low- and very low-quality evidence, the remaining medium- and high-quality recommendations were summarized and analyzed, resulting in 16 recommendations for TCM drug therapies, which were mainly from three guidelines (guideline numbers 2, 3 and 5). The target population of the recommendations is mainly divided into three categories: population susceptible to osteoporosis (osteopenia patients), patients with osteoporosis, and patients with osteoporotic fracture (OPF), the most common complication of osteoporosis. Patients with osteoporosis include primary osteoporosis (POP), postmenopausal osteoporosis (PMOP) and senile osteoporosis (SOP). Specific recommended drugs and outcome measures are detailed in **Tables 4**, **5**.

**Table 4:** Drug recommendations for osteoporosis patients.

Popul-ation(P)	Intervention(I)	Comparator(C)	Outcome(O)	GRADE (GN)
POP	Xianling Gubao Capsule +WM	WM	BMD (LV[MD=0.07,95%CI(0.05,0.08)])	1B(3)
	Gusongbao Capsule	Other Chinese patent medicines	BPSS ([MD=-1.18,95%CI(-1.67,-0.69)])	2B(3)
	Qianggu Capsule	WM, Gusongbao Capsule	BPSS ([MD=-1.00,95%CI(-1.50,-0.51)])	2B(3)
	Epimedium Total Flavonoid Capsule	Gushukang Capsule	BMD (LV[MD=0.02,95%CI(-0.03,0.07)])	2B(3)
SOP	Xianling Gubao Capsule	WM	BMD(LV[MD=0.03,95%CI(-0.00,0.06)], [MD=0.08,95%CI(0.06,0.10)])	2B(3,5)
	Xianling Gubao Capsule +WM		BMD (LV[MD=0.10,95%CI(0.08,0.12)], [MD=0.06,95%CI(0.05,0.07)])	2B(3,5)
			VAS ([MD=-1.54,95%CI(-2.40,-0.68)])	2B(5)
			BGP ([MD=4.09,95%CI(3.20,4.98)])	
			BALP ([MD=7.53,95%CI(5.91,9.14)])	
			S-Ca ([MD=0.03,95%CI(0.01,0.06)])	
			S-P ([MD=0.03,95%CI(0.00,0.05)])	
PMOP	Erxian Decoction	WM	CE ([OR=7.68,95%CI(1.67,35.38)])	1B(2)
			BMD ([MD=0.02,95%CI(0.02,0.03)])	
	Erxian Decoction +WM	WM	CE ([OR=4.02,95%CI(2.34,6.92)])	1B(2)
			BMD(LV[MD=0.05,95%CI(0.02,0.08)],FN [MD=0.04,95%CI(0.01,0.08)])	
	Jiawei Erxian Decoction +WM		CE ([OR=2.88,95%CI(1.20,6.90)])	1B(2)
	Xianling Gubao Capsule		BMD (LV[MD=-0.00,95%CI(-0.01,0.02)])	1B(3)
	Xianling Gubao Capsule +WM		BMD (LV[MD=0.07,95%CI(0.04,0.09)])	2B(3)
	Jintiange Capsule +WM		BMD (LV[MD=0.04,95%CI(0.01,0.07)])	2B(3)
			TCMSS([MD=-0.96,95%CI(-1.23,-0.69)],[MD=-0.8,95%CI(-1.13,-0.47)], [MD=-0.58,95%CI(-0.90,-0.26)])	1B(3)
	Qigu Capsule	Xianling Gubao Capsule	BMD (LV[MD=0,95%CI(-0.02,0.02)], FN[MD=0,95%CI(-0.02,0.01)])	2B(3)

Western Medicine, Western medicine basic treatment of osteoporosis, including calcium, active vitamin D and its analogs, bisphosphonates (including alendronate sodium, zoledronic acid, etc.), calcitonin and so on.

WM, Western Medicine; BPSS, Bone Pain Symptom Score; BMD, Bone Mineral Density; LV, Lumbar Vertebra; VAS, Visual Analogue Pain Scale; BGP, Bone-γ-Carboxyglutamic Acid-Containing Protein; BALP, Bone Alkaline Phosphatase; S-Ca, Serum Calcium; S-P, Serum Phosphate; CE, Clinical Efficacy; FN, Femoral Neck; TCMSS, Traditional Chinese Medicine Symptom Score.

**Table 5:** Drug recommendations for osteopenia patients and patients with osteoporotic fracture.

Population(P)	Intervention(I)	Comparator(C)	Outcome(O)	GRADE (GN)
Osteopenia	Qianggu Capsule	Gusongbao Capsule	BPSS ([MD=-1.32,95%CI(-2.05,-0.59)])	2B(3)
			TCMSS ([MD=-2.15,95%CI(-3.41,-0.89)])	1B(3)
OPF	Xianling Gubao Capsule +WM	WM	BMD(LV[MD=0.20,95%CI(0.15,0.24)], LV[MD=0.06,95%CI(0.04,0.08)])	2B(3)
	Xianling Gubao Capsule +WM		Cobb angle([MD=-2.98,95%CI(-3.53,-2.43)])	2B(3)
			ODI ([MD=-0.92,95%CI(-1.33,-0.51)], [MD=-8.04,95%CI(-9.45,-6.63)], [MD=-5.03,95%CI(-7.04,-3.02)])	

Western medicine, Western medicine basic treatment of osteoporosis, Including calcium, active vitamin D and its analogs, bisphosphonates (including alendronate sodium, zoledronic acid, etc.), calcitonin and so on.

WM, Western Medicine; BPSS, Bone Pain Symptom Score; TCMSS, Traditional Chinese Medicine Symptom Score; BMD, Bone Mineral Density; LV, Lumbar Vertebra; ODI, Oswestry Disability Index.

## 4 Discussion

### 4.1 Quality analysis

#### 4.1.1 Progress of osteoporosis guidelines on TCM drug therapies

The number of TCM drug therapy guidelines has been increasing steadily, with the initial appearance in 2006 and a slower growth rate until 2020. From 2020 to 2023, the number of guidelines doubled, reflecting a growing emphasis on standardized diagnosis and treatment by medical professionals. Stratified analysis shows that the quality of guidelines has improved over time, with post-2020 guidelines scoring significantly higher in “scope and purpose”, “stakeholder involvement”, and “editorial independence” domains. This suggests that developers have recognized previous deficiencies and implemented improvements.

In addition, the introduction of evidence systems has significantly enhanced guideline quality. Guidelines with evidence systems generally exhibited higher overall quality compared to those without, and all were classified as A or B grade guidelines. They ranked higher in “stakeholder involvement”, “rigor of development”, “clarity of presentation”, and “applicability” domains. Guidelines with evidence-based systems are more focused on guideline methodology than consensus guidelines and can significantly improve the scientificity, transparency, and applicability of guidelines.

Based on the comprehensive quality assessment, guidelines scored highly in the “scope and purpose” and “clarity of presentation” domains, with low variation. This indicated that the guidelines included in this study were particularly focused more on these two domains. Furthermore, these two domains emphasized standardization of guideline writing, indicating a higher level of standardization in the composition of osteoporosis guidelines on TCM drug therapies.

#### 4.1.2 The main deficiencies and targeted measures of the current osteoporosis guidelines on TCM drug therapies

This study included 20 guidelines, of which 17 were from China and only 3 were international guidelines. To a certain extent, this reflected the limited international recognition of TCM drug therapies for osteoporosis. Thus, it is necessary to further promote the development of international guidelines for TCM drug therapies.

At the same time, of the 20 guidelines, 12 are C-level guidelines, more than half. These low-quality guidelines may have potentially impacted the adoption and endorsement of TCM recommendations. Our comprehensive quality assessment revealed that both the overall guidelines and the C-level guidelines obtained low scores in the domains of “stakeholder involvement”, “rigor of development”, “applicability”, and “editorial independence”. Notably, the C-level guidelines scored even lower than the overall guidelines in these domains, particularly in the “rigor of development” domain. This pattern implies that the absence of C-level guidelines in these domains may be influencing the overall quality of guidelines in these areas.

Therefore, in order to enhance the overall quality of the guidelines, we propose targeted measures in these four domains: (1) Stakeholder involvement: the guidelines should furnish detailed information about the expert group members, especially regarding their role in guideline development and research fields. (2) Rigor of development: guideline developers should incorporate evidence and recommendation systems, detail literature search strategies and inclusion criteria, and elaborate on the specific methods used to formulate recommendations. Guidelines should undergo external reviews before publication and provide an explicit update process (including method, timing, and frequency). (3) Applicability: during guideline development, potential implementation obstacles should be fully considered; effective and explicit implementation tools should be provided, and the cost of implementing the recommendations should be thoroughly evaluated. (4) Editorial independence: the development organization should disclose any influence from sponsors and conflicts of interest among members to ensure the objectivity of the guidelines.

### 4.2 Analysis of drug recommendations results

#### 4.2.1 Active ingredients and mechanisms of action of TCM

In the guideline, we recommended six Chinese patent medicines and two decoctions. TCM are characterized by their synergistic effects on multiple pathways, targets, and signaling pathways through various active ingredients. The mechanism of action against osteoporosis includes: 1. inhibiting osteoclast resorption activity (31–33); 2. promoting bone trabecular maturation and osteoblast increase (34–39) (32, 40, 41); 3. accelerating osteoclast metabolic activity (42); 4. protecting gonads and increasing sex hormone levels; restoring the amount of bone lost due to the decline in the level of sex hormones (43, 44). We took the representative traditional Chinese patent medicine (Xianling Gubao capsule) and decoction (Erxian decoction) with high recommendation frequency and many research materials as examples to further analyze their active ingredients and mechanism of action.

Xianling Gubao capsule: The flavonoids in the core active ingredients increase BMD, bone volume/tissue volume (BV/TV), trabecular number (Tb.N), and trabecular thickness (Tb.Th), and decrease bone surface/bone volume (BS/BV) in osteoporotic rats; regulate the expression of osteoprotegerin (OPG) and receptor activator of nuclear factor-κB(RANKL) proteins, thereby inhibiting osteoclast generation, decreasing bone resorption, and inhibiting the development of osteoporosis (33). Furthermore, key active ingredients such as psoralen, isostatin, and sulforaphane activate adenylate cyclase to promote the biosynthesis of various sex steroid hormones, elevating the levels of 17β-estradiol, luteinizing hormone, 12α-hydroxyprogesterone, and androstenedione (43). Capsule also improves the bone growth factor bone morphogenetic protein 2(BMP-2) expression in osteoporotic rats with fractures, enhances insulin-like growth factor-1 (IGF-1) expression, and promotes bone metabolism, scab formation, bone density, biomechanics, and fracture healing (42).

Erxian Decoction (EXD): The main active ingredients are Monotropein, mangiferin, berberine hydrochloride, ferulic acid, curculigoside, and icariin (45). EXD exerts estrogen-like effects and is effective in reducing bone loss by increasing BMD and improving bone microarchitecture as well as restoring serum levels of the osteoblast-secreted (OCN) protein (44); it also enhances the proliferation rate of osteoblast-like UMR-0 cells in rats (46), and promotes the proliferation of mouse embryonic osteoblast precursor cells, Mc3t3-e1 cells, and osteogenic differentiation by regulating the BK channel (47). EXD also activates the Insulin-like growth factor 1 receptor/Phosphatidylinositol 3-kinase/Protein kinase B(IGF1/PI3K/AKT) signaling pathway by increasing serum IGF1 concentration and tibial Insulin-like growth factor 1 receptor(IGF1R), PI3K, and AKT expression, thereby promoting osteogenic differentiation and proliferation of osteoblasts. Moreover, it regulates the concentration of medium and long-chain free fatty acid (MLCFA) and inhibits Stearoyl-CoA desaturase 1 (SCD1) activity, restoring disturbed lipid metabolism in adipose tissue and promoting fatty acid synthetase expression through activation of the IGF1/PI3K/AKT signaling pathway to alleviate osteoporosis symptoms (45).

#### 4.2.2 Clinical efficacy of TCM

The TCM in the recommended guidelines had several clinical randomized controlled trials (RCTs) to verify its efficacy. The main clinical outcome of the clinical studies is the BMD value, combined with bone pain score, clinical effectiveness rate and osteocalcin, blood calcium and phosphorus. Systematic evaluation and evidence quality grading showed that the quality of evidence was moderate.

For example, in the case of Xianling Gubao capsules, five RCTs (48–52) for the treatment of patients with POP were conducted, with a total sample size of 610 cases in Meta-analysis (304 cases in the experimental group and 306 cases in the control group). The largest sample size in a single clinical trial was 192 cases, and the smallest sample size was 60 cases. The results showed that the use of TCM alone can significantly increase the BMD values of patients. For studies on the treatment of patients with SOP, a meta-analysis of 3 RCTs (53–55) was conducted, with a total sample size of 292 cases (146 cases in the test group and 146 cases in the control group). The largest sample size in a single clinical trial was 160 cases, and the smallest sample size was 64 cases. The results showed that the use of TCM alone could significantly increase the BMD values of the patients; furthermore, a Meta-analysis (56) of 54 clinical studies (including 22 RCTs) was conducted, with a total sample size of 5110 cases (2583 cases in the test group and 2527 cases in the control group). The largest sample size in a single clinical trial was 300 cases, and the smallest sample size was 50 cases. The results showed that the combination of TCM and Western medicine could significantly improve patients’ bone mineral density values, VAS for pain, and levels of alkaline phosphatase, osteocalcin, and blood calcium and phosphorus. For studies on the treatment of patients with PMOP, a meta-analysis of 2 RCTs (57, 58) was concluded, with a total sample size of 699 cases (231 cases in the experimental group and 468 cases in the control group). The largest sample size in a single clinical trial was 474 cases, and the smallest sample size was 225 cases. The results showed that using TCM alone can significantly improve the BMD values of patients. A meta-analysis of the 2 RCTs (59, 60) was concluded, with a total sample size of 304 cases (152 cases in the experimental group and 152 cases in the control group). The largest sample size in a single clinical trial was 200 cases, and the smallest sample size was 104 cases. The results showed that the combination of TCM and Western medicine could significantly improve the BMD values of patients. After checking the original studies, all the above RCT trial groups were comparable with the control group at baseline (48–60). All used objective BMD as the primary clinical outcome with high stability, confidence, and comparability (48–54, 56–60). Two RCTs (57, 58) utilized a double-blind design.

In summary, the basic studies confirmed that multiple active ingredients of TCM exert synergistic effects of multiple pathways, targets, and signaling pathways in the prevention and treatment of osteoporosis. The level of clinical research evidence is moderate, with some shortcomings in trial design and control of confounding factors. More high-quality and rigorous clinical observational studies are still needed. Several clinical RCT have demonstrated that the use of TCM alone or in combination with Western medicine can improve patients’ bone density values and improve clinical symptoms. From a pharmacoeconomic perspective, TCM is more cost-effective when the individual willingness-to-pay threshold meets certain requirements (61, 62).

## Data availability statement

The original contributions presented in the study are included in the article/supplementary material. Further inquiries can be directed to the corresponding author.

## Author contributions

LZ: Writing – original draft. JL: Writing – original draft. RX: Writing – review & editing, Writing – original draft. LFZ: Writing – review & editing, Writing – original draft. WC: Writing – original draft. HL: Writing – original draft.
