# Supplementary material for: Osteoporosis guidelines on TCM drug therapies: a systematic quality evaluation and content analysis
Source: Front Endocrinol (Lausanne). 2024 Jan 22;14:1276631. doi: 10.3389/fendo.2023.1276631 (PMC10839061; doi:10.3389/fendo.2023.1276631)
Supplement: Supplementary file 1 [file Table_1.docx]

**PubMed（3）**

#1 ("Guidelines as Topic"[MeSH Major Topic]) OR (guideline*[Title]) OR (guidance*[Title]) OR (recommendation*[Title]) OR (consensus*[Title]) OR (statement*[Title])

#2 "Practice Guideline" [Publication Type] OR "Guideline" [Publication Type]

#3 #1 OR #2

#4 ((((("Osteoporosis"[Mesh]) OR Bone Loss[Title/Abstract]) OR Bone Losses[Title/Abstract]) OR Osteoporosis[Title/Abstract]) OR Osteoporoses[Title/Abstract])

#5 #3 AND #4

#6 (“medicine, Chinese Traditional”[MeSH Major Topic]) OR (“Herbal Medicine”[MeSH]) OR ("Traditional Chinese medicine"[Text Word]) OR ("Chinese medicine"[Text Word]) OR ("Chinese herbal"[Text Word]) OR ("TCM"[Text Word]) OR ("botanical*"[Text Word]) OR ("herb*"[Text Word]) OR (“Herbal Medicine”[Text Word]) OR ("Natural Medicine"[Text Word])

#7 #5 AND #6 Filters: Full text


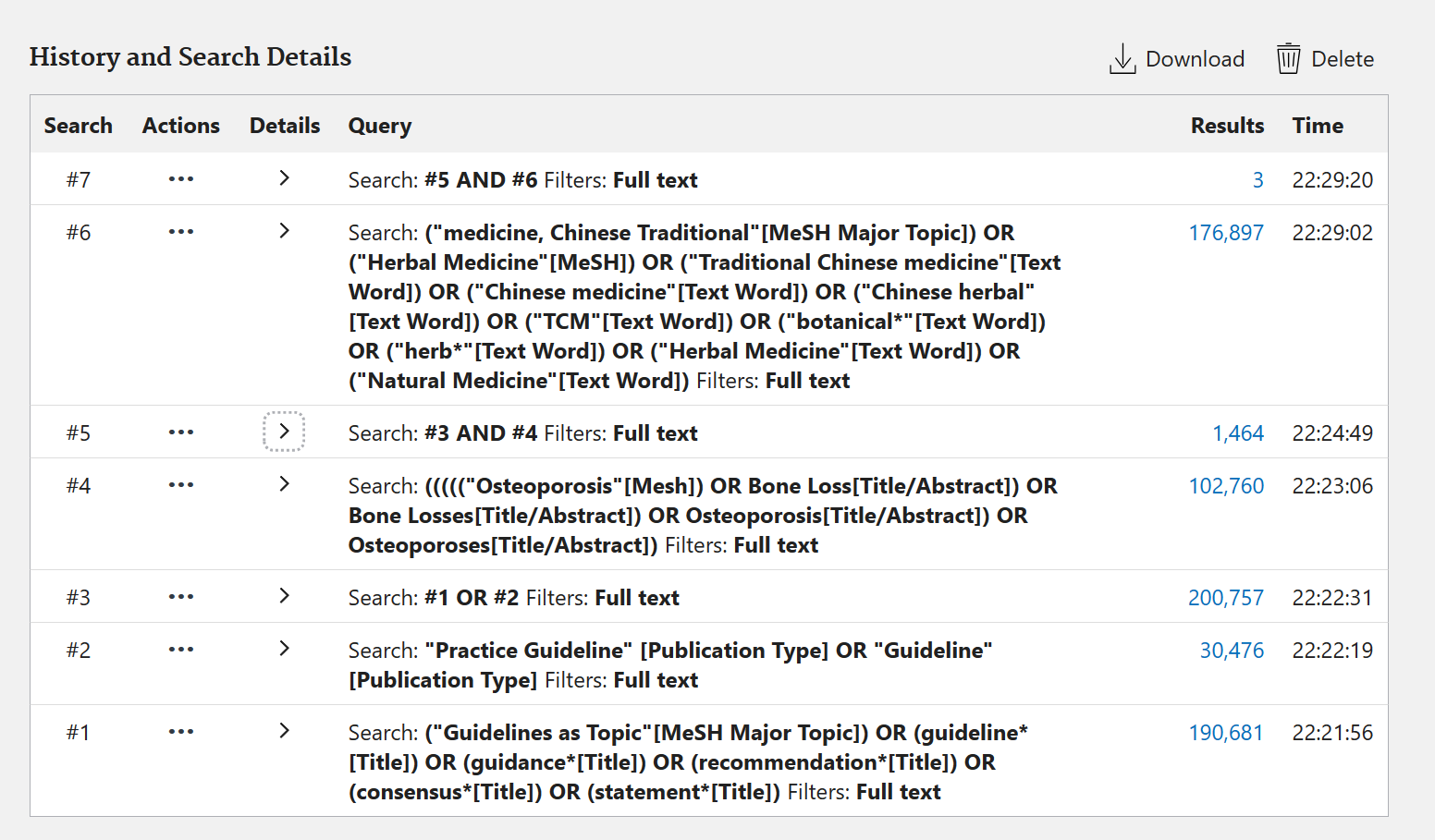


**Embase（47）**

#1 guideline*:ti OR guidance*:ti OR recommendation*:ti OR consensus*:ti OR statement*:ti OR 'practice guideline'/exp

#2 'osteoporosis'/exp OR 'Bone Loss':ti,ab,kw OR 'Bone Losses':ti,ab,kw OR 'Osteoporosis':ti,ab,kw OR 'Osteoporoses':ti,ab,kw

#3 #1 AND #2

#4 'Traditional Chinese medicine':ti,ab,kw OR 'Chinese medicine':ti,ab,kw OR 'Chinese herbal':ti,ab,kw OR 'TCM':ti,ab,kw OR 'botanical*':ti,ab,kw OR 'herb*':ti,ab,kw OR 'Herbal Medicine':ti,ab,kw OR 'Natural Medicine':ti,ab,kw OR 'chinese medicine'/exp OR 'Herbal Medicine'/exp

#5 #3 AND #4


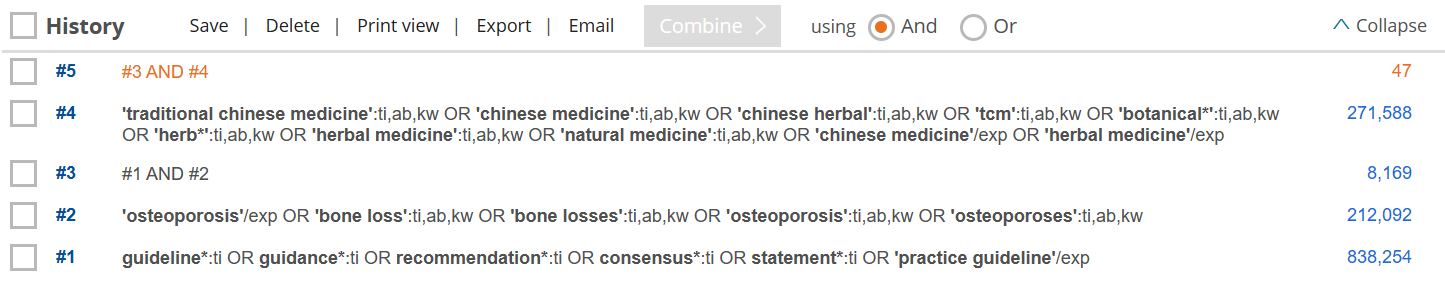


**Web of science（8）**

#1 (((((TI=(guideline*)) OR TI=(guidance*)) OR TI=(recommendation*)) OR TI=(consensus*)) OR TI=(statement*))

#2 ((((TS=("Bone Loss")) OR TS=("Bone Losses")) OR TS=("Osteoporosis")) OR TS=("Osteoporoses"))

#3 #2 AND #1

#4 ((((((((TS=("Traditional Chinese medicine")) OR TS=("Chinese medicine")) OR TS=("Chinese herbal")) OR TS=("TCM")) OR TS=("botanical*")) OR TS=("herb*")) OR TS=("Herbal Medicine")) OR TS=("Natural Medicine"))

#5 #3 AND #4 and 预印本 (排除 – 数据库)


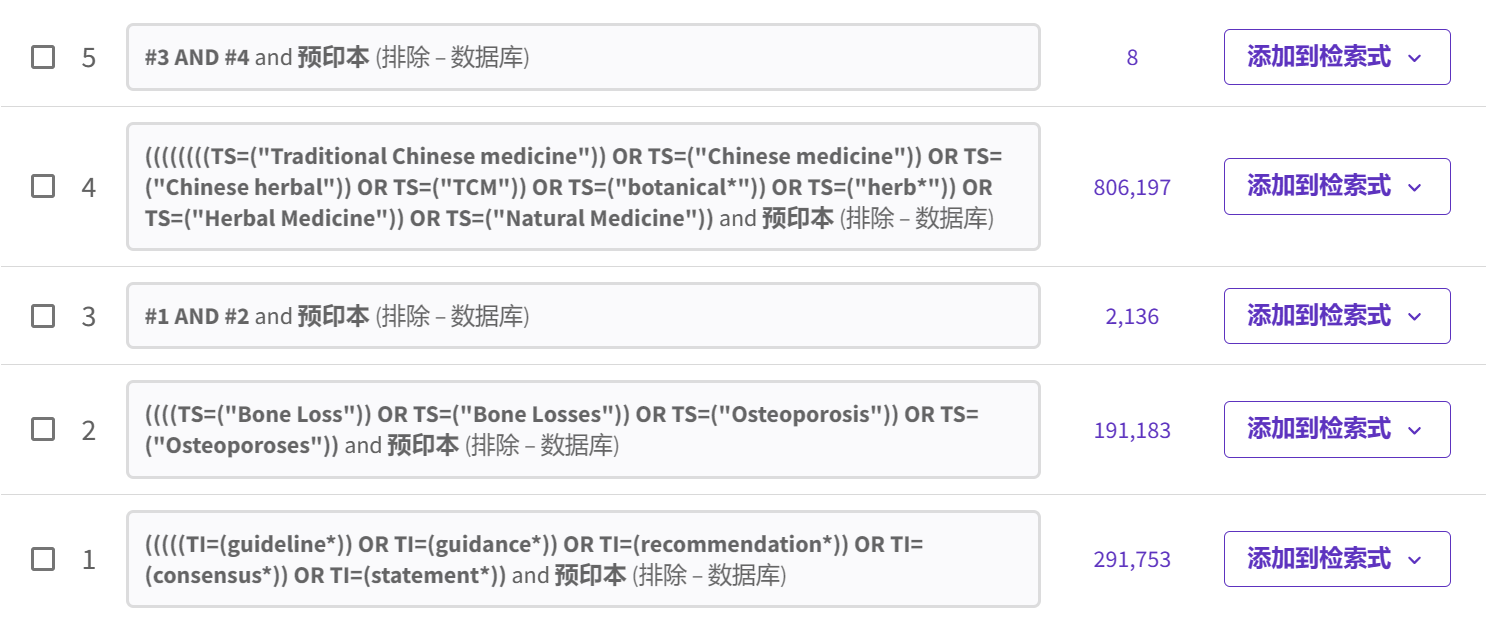


**CNKI（126）**

#1 TI='指南'+'指引'+'推荐'+'共识'+'声明'

#2 FT='中医'+'中医药'+'中药'+'中西医'+'中成药'+'传统医学'+'草药'+'植物药'+'天然药物'

#3 FT='骨质疏松'+'骨质减少'

#5 #1 and #2 and #3 and #4


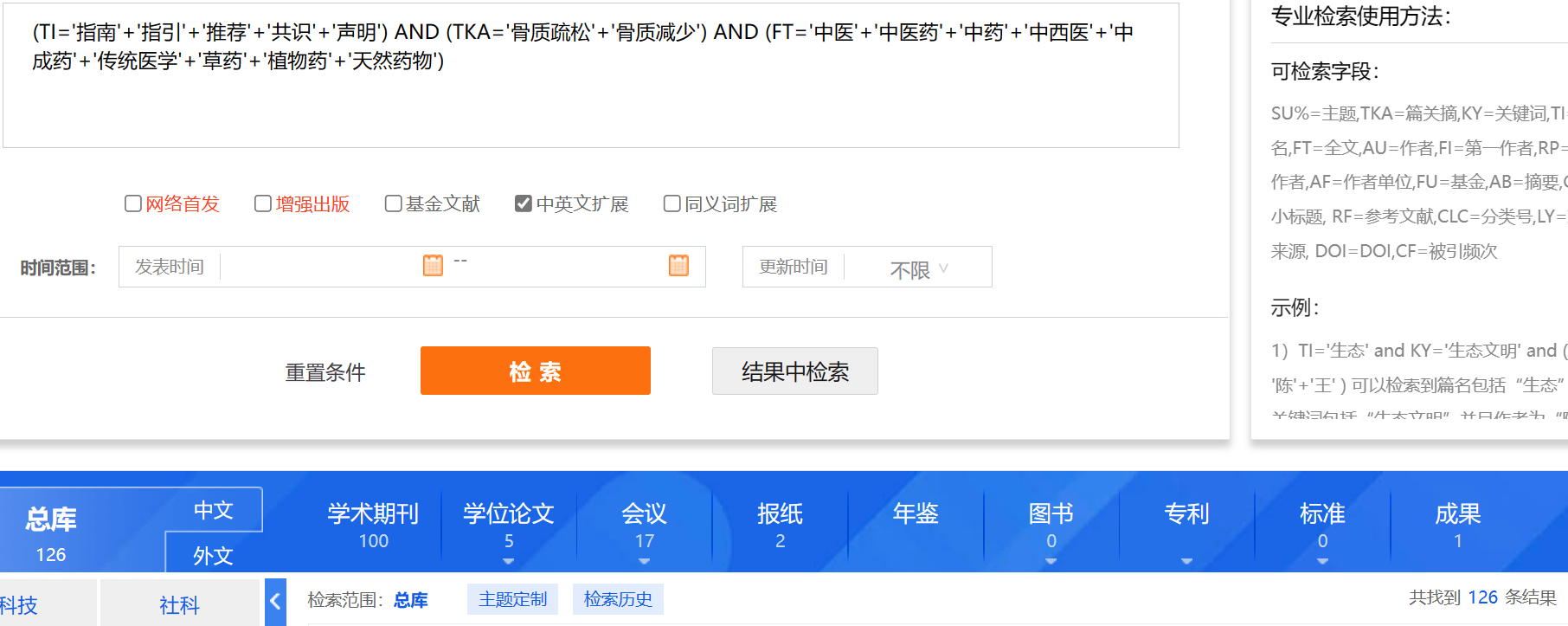


**VIP（22）**

#1 题名:("指南" or ”指引” or ”推荐” or "共识" or ”声明”)

#2 全部:(“中医” or ”中医药” or ”中药” or ”中西医” or ”中成药” or “传统医学” or “草药” or “植物药” or “天然药物”)

#3 全部:("骨质疏松" or "骨质减少")

#4 #1 and #2 and #3


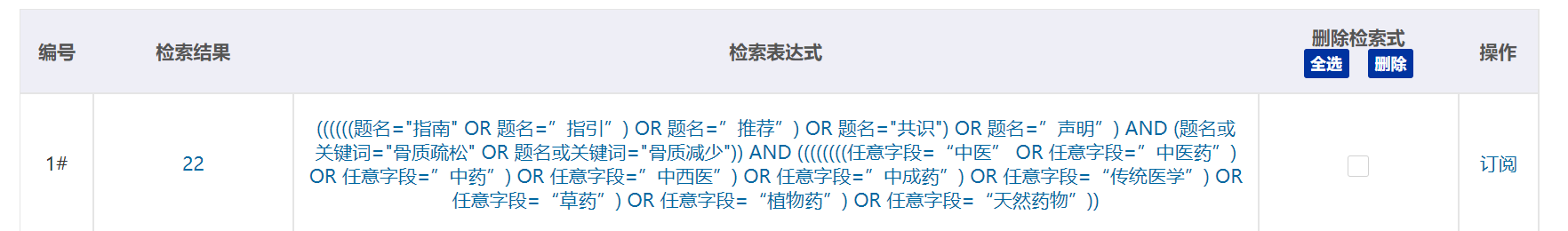


**Wangfang（57）**

#1 T=("指南" or ”指引” or ”推荐” or "共识" or ”声明”)

#2 M=("骨质疏松" or "骨质减少")

#3 U=(“中医” or ”中医药” or ”中药” or ”中西医” or ”中成药” or “传统医学” or “草药” or “植物药” or “天然药物”)

#4 #1 and #2 and #3

T=("指南" or ”指引” or ”推荐” or "共识" or ”声明”) and M=("骨质疏松" or "骨质减少") and U=(“中医” or ”中医药” or ”中药” or ”中西医” or ”中成药” or “传统医学” or “草药” or “植物药” or “天然药物”)


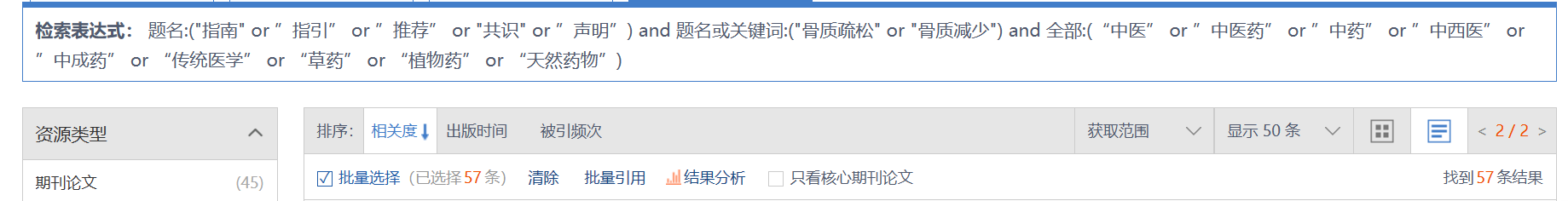


**CBM（52）**

#1 "指南"[标题:智能] OR "指引"[标题:智能] OR "推荐"[标题:智能] OR "共识"[标题:智能] OR "声明"[标题:智能] OR "指南"[不加权:扩展]

#2 "骨质疏松"[不加权:扩展] OR "骨质疏松"[常用字段:智能] OR "骨质减少"[常用字段:智能]

#3 "中医"[全部字段:智能] OR "中医药"[全部字段:智能] OR "中药"[全部字段:智能] OR "中西医"[全部字段:智能] OR "中成药"[全部字段:智能] OR "传统医学"[全部字段:智能] OR "草药"[全部字段:智能] OR "植物药"[全部字段:智能] OR "天然药物"[全部字段:智能] OR "医学, 中国传统"[不加权:扩展] OR "中草药"[不加权:扩展] OR "植物药"[不加权:扩展] OR "中成药"[不加权:扩展] OR "天然药材"[不加权:扩展]

#4 #1 AND #2 AND #3


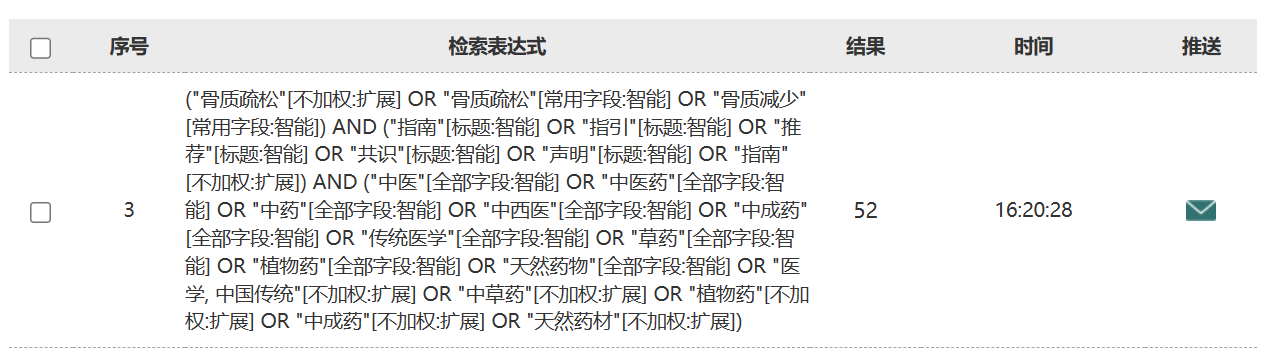


**Guideline website (41)：**

Search terms: Osteoporosis

National Guideline Clearinghouse, NGC ([Guidelines and Measures | Agency for Healthcare Research and Quality (ahrq.gov)](https://www.ahrq.gov/gam/index.html))

1. Long-Term Drug Therapy and Drug Holidays for Osteoporotic Fracture Prevention

2. Screening for Osteoporosis U.S. Preventive Services Task Force Recommendation Statement

National Institute for Health and Clinical Excellence, NICE ([Published guidance, NICE advice and quality standards | Guidance | NICE](https://www.nice.org.uk/guidance/published?q=osteoporoses))

*No records searched*

Scottish Intercollegiate Guidelines Network, SIGN ([Our guidelines (sign.ac.uk)](https://www.sign.ac.uk/our-guidelines/))

3. Sign-142-osteoporosis-v3

Guidelines International Network, GIN ([Guidelines International Network | Guidelines International Network (GIN) (ebmportal.com)](https://guidelines.ebmportal.com/guidelines-international-network?search=Bone%20Loss&type=search))

4. CPG Mx of Osteoporosis Second Edition (2015)

5. KNGF Guideline for Physical Therapy in patients with Osteoarthritis of the hip and knee

6. KNGF Guideline for Physical Therapy in patients with Osteoporosis

7. Management of Osteoporosis in Survivors of Adult Cancers With Nonmetastatic Disease ASCO Clinical Practice Guideline

8. Osteoporosis assessing the risk of fragility fracture (CG146)

9. Pharmacological Management of Osteoporosis in Postmenopausal Women An Endocrine Society Guideline Update

10. Sign-142-osteoporosis-v3

11. Treatment of Low Bone Density or Osteoporosis to Prevent Fractures in Men and Women A Clinical Practice Guideline Update From the American College of Physicians

WHO

*No records searched*

Medlive ([临床指南_临床诊疗指南_医脉通 (medlive.cn)](https://guide.medlive.cn/))

12. 原发性骨质疏松症诊疗指南(+2017)

13. 中医药防治原发性骨质疏松症专家共识（2015）

14. 男性骨质疏松症诊疗指南

15. 原发性骨质疏松症诊疗指南（2022）

16. 中国老年骨质疏松症诊疗指南（2018）

17. 中医药防治原发性骨质疏松症专家共识(2020)

18. 基层医疗机构骨质疏松症诊断和治疗专家共识（2021）

19.2013 OSHK Guideline for Clinical Management of Postmenopausal Osteoporosis in Hong Kong

20. Guidelines on the management and treatment of glucocorticoid-induced osteoporosis of the Japanese Society for Bone and Mineral Research: 2014 update

21. Osteoporosis in Menopause

22. Italian association of clinical endocrinologists (AME) position statement: drug therapy of osteoporosis

23.2017 American College of Rheumatology Guideline for the Prevention and Treatment of Glucocorticoid-Induced Osteoporosi

24. Treatment of Low Bone Density or Osteoporosis to Prevent Fractures in Men and Women: A Clinical Practice Guideline Update from the American College of Physicians

25. Guidelines of the Brazilian Society of Rheumatology for the diagnosis and treatment of osteoporosis in men

26. Clinical guidelines for the prevention and treatment of osteoporosis: summary statements and recommendations from the Italian Society for Orthopaedics and Traumatology

27.2018 Update of French Recommendations on the Management of Postmenopausal Osteoporosis

28. The prevention of glucocorticoid-induced osteoporosis in patients with immune thrombocytopenia receiving steroids: a British Society for Haematology Good Practice Paper

29.Pharmacological Management of Osteoporosis in Postmenopausal Women: An Endocrine Society Guideline Update

30. Management of osteoporosis in postmenopausal women: the 2021 position statement of The North American Menopause Society

31. Management of Postmenopausal Osteoporosis

32. Dietary Recommendations in the Prevention and Treatment of Osteoporosis

33. French recommendations on the prevention and treatment of osteoporosis secondary to bariatric surgery

34. Best Practice Guidelines for Assessment and Management of Osteoporosis in Adult Patients Undergoing Elective Spinal Reconstruction

35. Pharmacologic Treatment of Primary Osteoporosis or Low Bone Mass to Prevent Fractures in Adults: A Living Clinical Guideline From the American College of Physicians

36. Prevention and Treatment of Glucocorticoid-Induced Osteoporosis in Adults: Consensus Recommendations From the Belgian Bone Club

37. 骨质疏松症康复指南

38. 糖皮质激素性骨质疏松症的诊疗规范

39. 原发性骨质疏松症患者的营养和运动管理专家共识

40. 骨质疏松症中西医结合诊疗专家共识

41. 肌少-骨质疏松症专家共识

**Google Scholar（4）**

1. AMERICAN ASSOCIATION OF CLINICAL ENDOCRINOLOGISTS/ AMERICAN COLLEGE OF ENDOCRINOLOGY CLINICAL PRACTICE GUIDELINES FOR THE DIAGNOSIS AND TREATMENT OF POSTMENOPAUSAL OSTEOPOROSIS— 2020 UPDATE

2. Guidelines for the management of osteoporosis and fragility fractures

3. European guidance for the diagnosis and management of osteoporosis in postmenopausal women

4. UK clinical guideline for the prevention and treatment of osteoporosis
